# Unusual Presentation of Gastric Perforation by Foreign Body: A Case Report

**DOI:** 10.1155/2011/509806

**Published:** 2011-10-19

**Authors:** V. S. R. Rao, R. Sarkar, Richard Turner, K. R. Wedgwood

**Affiliations:** Department of General Surgery, Castle Hill Hospital, Castle Road, Cottingham HU16 5BQ, UK

## Abstract

Perforation of the gastrointestinal tract by ingested foreign body is rare. The majority of patients do not recall ingestion of the foreign body, and dietary foreign bodies are most commonly involved. We present an interesting case where the offending foreign body gave rise to a diagnostic dilemma masquerading as a pancreatic mass. A high index of suspicion is indicated especially when dealing with atypical presentation and nonspecific symptoms as highlighted in this case.

## 1. Introduction

Ingestion of foreign body is not an uncommon occurrence. The vast majority pass through the gastrointestinal tract uneventfully, and perforation is rare. We report an unusual presentation of a gastric perforation by a foreign body and discuss various issues regarding investigation and management.

## 2. Case Presentation

A 62-year-old man presented with history of dysphagia to solids, night sweats, lethargy, and significant weight loss of 2 stones over 2 months. Apart from dyspepsia on symptomatic treatment, he did not have any significant comorbidities. He worked as a chef on ships and was recently admitted overseas with an episode of sun stroke for which he was hospitalised for a week.

**Figure 1:**
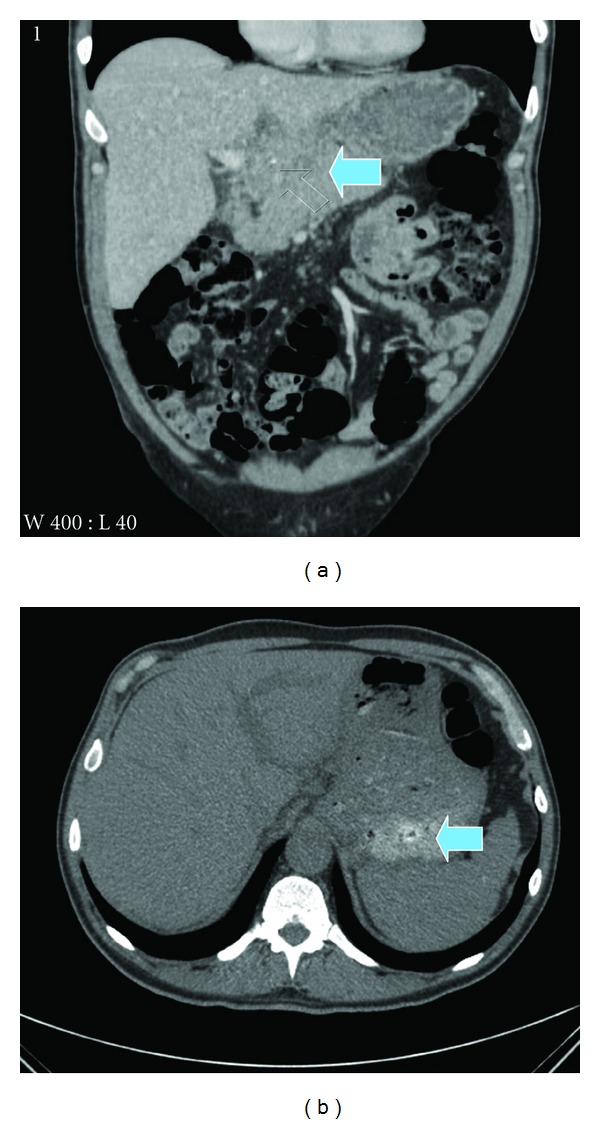
CT scan revealing a complex soft tissue mass in relation to the pancreas with a calcified focus.

On clinical examination, he had fullness in the epigastrium with minimal tenderness. Blood tests revealed leucocytosis and elevated C reactive protein with mildly deranged liver function tests characterised by raised alkaline phosphatase and lactate dehydrogenase levels. Tumour markers including CEA and CA19.9 were normal. Upper gastrointestinal endoscopy revealed inflamed and oedematous posterior gastric wall, and biopsies were positive for H. pylori infestation. Ultrasound revealed a 5 cm heterogenous mass in close relation to the head of pancreas with associated mild intra- and extrahepatic biliary dilatation. CT scan confirmed a 6 × 6 cm soft tissue mass in the pancreatic bed with a 4 cm linear calcified mass (Figure 1). There was associated mesenteric lymphadenopathy with thickening of the posterior gastric wall. Differential diagnosis entertained included lymphoma, gastric cancer, and possible foreign body in view of the calcified mass.

In view of diagnostic ambiguity, exploratory laparotomy was undertaken. At laparotomy, a large inflammatory mass which appeared to arise from the duodenum and in close relation to the stomach, pancreas, and the liver was encountered. The initial impression was that this was probably a lymphoma. However, on further dissection, an abscess cavity in the pancreatic bed with a 4 cm foreign body was found. This was sent for histology and a drain was placed in the abscess cavity. The patient made an uneventful recovery and was discharged home within a week. 

Histology revealed soft tissue consistent with inflammatory granulation tissue, and the foreign body was reported to have haversian systems consistent with bony remodelling not found in modern bony fish and hence probably a chicken bone.

The patient recovered well and regained all lost weight. On further history review, he denied any history of foreign body or bone ingestion.

## 3. Discussion

Ingested foreign body perforation in the adult population is usually secondary to involuntary accidental ingestion and is frequently caused by dietary foreign bodies [1]. Predisposing factors include patients with dentures, patients with history of psychosis and alcohol abuse, and jail inmates [2]. More often, these include tooth picks, dentures, and dietary foreign bodies such as fish bone and chicken bone. A definite preoperative history of foreign body ingestion is rare as highlighted in the above case. Common sites include distal ileum, sigmoid colon, or rectum. Patients with foreign body perforation in the stomach, duodenum, and large bowel are more likely to present with longer, more innocuous clinical picture with chronic symptoms such as abdominal mass or abscess and are usually apyrexial with normal white cell count when compared to those with perforation in the small bowel [1]. The patient described in this report was admitted briefly prior to this presentation with what was thought to be sun stroke and treated with antibiotics. Closer scrutiny of the history revealed that he had chills and rigor with abdominal pain and this might have been due to gastric perforation which gave rise to the abscess in the lesser sac. This highlights the fact that perforation of the stomach and duodenum by foreign bodies might have an indolent course rather than an acute presentation. Other modalities of presentation include acute abdomen of uncertain origin, acute appendicitis, or acute diverticulitis [3]. Rare presentations include liver abscess secondary to migration of foreign bodies from the gut [4, 5].

## References

[1] Goh BKP, Chow PKH, Quah HM (2006). Perforation of the gastrointestinal tract secondary to ingestion of foreign bodies. *World Journal of Surgery*.

[2] Velitchkov NG, Grigorov GI, Losanoff JE, Kjossev KT (1996). Ingested foreign bodies of the gastrointestinal tract: retrospective analysis of 542 cases. *World Journal of Surgery*.

[3] Rodríguez-Hermosa JI, Codina-Cazador A, Sirvent JM, Martín A, Gironès J, Garsot E (2008). Surgically treated perforations of the gastrointestinal tract caused by ingested foreign bodies. *Colorectal Disease*.

[4] Kadowaki Y, Tamura R, Okamoto T, Mori T, Mori T (2007). Ruptured hepatic abscess caused by fish bone penetration of the duodenal wall: report of a case. *Surgery Today*.

[5] Santos SA, Alberto SCF, Cruz E (2007). Hepatic abscess induced by foreign body: case report and literature review. *World Journal of Gastroenterology*.

